# “Why Didn’t it Work?” Lessons From a Randomized Controlled Trial of a Web-based Personally Controlled Health Management System for Adults with Asthma

**DOI:** 10.2196/jmir.4734

**Published:** 2015-12-15

**Authors:** Annie YS Lau, Amaël Arguel, Sarah Dennis, Siaw-Teng Liaw, Enrico Coiera

**Affiliations:** ^1^Centre for Health InformaticsAustralian Institute of Health InnovationMacquarie UniversitySydneyAustralia; ^2^ARC-SRI Science of Learning Research Centre, Macquarie UniversitySydneyAustralia; ^3^Discipline of Physiotherapy, University of SydneySydneyAustralia; ^4^Centre for Primary Health Care and Equity, School of Public Health & Community Medicine, UNSW Medicine, University of New South WalesSydneyAustralia

**Keywords:** asthma, self-management, personal health record, personally controlled health management system, eHealth, Internet, intervention, adult

## Abstract

**Background:**

Personally controlled health management systems (PCHMS), which may include a personal health record (PHR), health management tools, and information resources, have been advocated as a next-generation technology to improve health behaviors and outcomes. There have been successful trials of PCHMS in various health settings. However, there is mixed evidence for whether consumers will use these systems over the long term and whether they ultimately lead to improved health outcomes and behaviors.

**Objective:**

The aim was to test whether use of a PCHMS by consumers can increase the uptake or updating of a written asthma action plan (AAP) among adults with asthma.

**Methods:**

A 12-month parallel 2-group randomized controlled trial was conducted. Participants living with asthma were recruited nationally in Australia between April and August 2013, and randomized 1:1 to either the PCHMS group or control group (online static educational content). The primary outcome measure was possession of an up-to-date written AAP poststudy. Secondary measures included (1) utilizing the AAP; (2) planned or unplanned visits to a health care professional for asthma-related concerns; (3) severe asthma exacerbation, inadequately controlled asthma, or worsening of asthma that required a change in treatment; and (4) number of days lost from work or study due to asthma. Ancillary analyses examined reasons for adoption or nonadoption of the intervention. Outcome measures were collected by online questionnaire prestudy, monthly, and poststudy.

**Results:**

A total of 330 eligible participants were randomized into 1 of 2 arms (intervention: n=154; control: n=176). Access to the PCHMS was not associated with a significant difference in any of the primary or secondary outcomes. Most participants (80.5%, 124/154) did not access the intervention or accessed it only once.

**Conclusions:**

Despite the intervention being effective in other preventive care settings, system use was negligible and outcome changes were not seen as a result. Consumers must perceive the need for assistance with a task and assign priority to the task supported by the eHealth intervention. Additionally, the cost of adopting the intervention (eg, additional effort, time spent learning the new system) must be lower than the benefit. Otherwise, there is high risk consumers will not adopt the eHealth intervention.

**Trial Registration:**

Australian New Zealand Clinical Trials Registry (ANZCTR): ACTRN12612000716864; https://www.anzctr.org.au/Trial/Registration/TrialReview.aspx?id=362714 (Archived by WebCite® at http://www.webcitation.org/6dMV6hg4A)

## Introduction

Personally controlled health management systems (PCHMS), which may include a personal health record (PHR), health management tools, and information resources, have been advocated as a next-generation technology to improve health behaviors and outcomes [1]. Trials of PCHMS have been undertaken in various health settings, including in vitro fertilization [2], hypertension [3], diabetes [4,5], influenza vaccination [6,7], sexually transmitted infection [8], medication accuracy [9], breast cancer management [10], and physical and emotional well-being [11,12]. However, there is mixed evidence for whether consumers will use these systems over the long term and whether they ultimately lead to improved health outcomes and behaviors [2-7,9-12].

Lack of engagement in digital interventions is a common phenomenon [13,14] and the reasons for adoption or nonadoption remain underreported. An early analysis of the reasons that led to the abandonment of a national PHR in the United Kingdom concluded that unless a system aligned closely with people’s attitudes, practices, information needs, and preexisting health services, then the risk it will not be adopted is substantial [15]. More recent analyses suggest that for chronic illnesses, PCHMS work best when there is a feedback loop between monitoring in the PHR and behaviors that could be self-managed by a consumer [16].

In this study, we examine how effective a PCHMS is in encouraging adults with a chronic condition—asthma—to obtain a written asthma action plan (AAP) from their primary care practitioner over a 12-month period. We also explored the reasons that underlay the adoption or nonadoption of the intervention.

### Asthma and the Written Asthma Action Plan

Asthma is a chronic condition [17,18]. The prevalence of asthma is significant; worldwide, the number of people suffering from asthma is approximately 300 million [17]. According to a Cochrane review, one of the most efficient tools that patients can use to manage their asthma is a written AAP [19]. The AAP is a set of instructions prepared with a health care professional that helps recognize signs that asthma is worsening, indicates which medication to use, or provides nonmedication strategies to keep asthma under control [20]. Written AAPs are individualized documents that must be updated (eg, once a year) by a clinician to match the evolution of the individual’s asthma condition [20].

When properly used, written AAPs are associated with fewer visits to the emergency department with an asthma exacerbation, fewer hospital admissions, better lung function, and an overall improvement of asthma symptoms [19]. Although having an up-to-date AAP is highly recommended, written AAPs are widely underused among adults; only 1 in 5 patients actually possess an up-to-date and usable AAP [21,22]. Most initiatives to improve the uptake of the written AAP have been targeted at health care professionals. Few have targeted patients.

This randomized controlled trial (RCT) is designed to test whether a PCHMS, tailored to help adults with asthma, would increase their rate of obtaining or updating a written AAP from a health care professional and whether this would lead to an improvement in asthma control.

### Hypotheses

Compared to participants allocated to the control group (ie, static online educational page), we hypothesized that those using a PCHMS are

1. More likely to obtain or update a written AAP;
2. More likely to make planned visits to a health care professional for asthma;
3. Less likely to make unplanned visits to a health care professional for asthma; and
4. Less likely to experience (1) severe asthma exacerbation, (2) inadequately controlled asthma, (3) worsening of asthma that requires a change in treatment, or (4) days lost from work or study due to asthma.

## Methods

Details on participants, recruitment strategy, intervention description, data collection, ethical considerations, and study procedure are described in the study protocol [23]. Utilization of AAP was defined by participant self-report to the questions “During the study, when you experienced an asthma exacerbation, did you use your written asthma action plan?” and “How often did you use your AAP during the study?”

### Trial Design

In this parallel 2-arm RCT, participants were stratified by gender and level of asthma severity (intermittent vs persistent), and randomized 1:1 to have immediate access to the PCHMS or to control.

Participant recruitment took place between April and August 2013. All individuals who expressed an interest were assessed with a 5-minute online eligibility questionnaire. Eligible individuals were then invited to complete a 10- to 15-minute prestudy questionnaire. Participants in both arms continued to receive usual care from their health services and were surveyed monthly for asthma symptoms, asthma exacerbation, asthma control, and other competing priorities, and followed up with a 10- to 15-minute poststudy questionnaire between April and June 2014.

### Participants and Setting

Eligible participants were adults (aged 18 years and older) living in Australia diagnosed with asthma, who had at least monthly access to the Internet and email, and had sufficient English language skills.

### Control Group

On completion of the prestudy questionnaire, participants who had been randomly allocated to the control arm were redirected to a static webpage with links to patient websites (eg, the Asthma Foundation, HealthInsite, myDR) that provided educational information on asthma. They were advised that they would be contacted to complete monthly surveys to elicit their asthma status during the study and would receive a follow-up questionnaire on conclusion of the study.

### Intervention Group

Full details of the Healthy.me Web-based PCHMS are described elsewhere [11,12,23]. During the study, Healthy.me provided participants with evidence-based information about asthma, the importance of a written AAP, and ways of obtaining a plan from a health care professional. Additionally, participants received monthly email reminders about the various interactive features of Healthy.me (eg, forum, poll, PHR).

An expert steering group was formed to tailor educational content for patients with asthma and to customize the interactive features of Healthy.me to deliver this content over the 12 months. Three asthma “journeys” were developed, providing evidence-based material to consumers about the written AAP. A usability study with 10 individuals was conducted and all major usability issues associated with the content and the intervention were addressed before commencing the study.

### Theoretical Framework of the Intervention

A review of online interventions found that those built on a theoretical framework demonstrated greater efficacy [24]. The Health Belief Model (HBM) [25], a prominent model of behavioral change, was used to guide the design of the 3 asthma journeys. More details are available in Multimedia Appendix 1.

There are strong theoretical reasons why the PCHMS features drive behavioral change:

1. The online appointment booking service, embedded within health service information descriptions, allows consumers to turn information into action in keeping with the “cue to action” elements of the HBM [25].
2. Social features (eg, polls and forums), which allow individuals to connect with others and observe social norms on health behaviors, are designed according to principles of social cognitive theory [26].
3. PHRs, which facilitate self-management and self-awareness, are related to the principle of increasing self-efficacy.
4. The journey model, which allows stages of change described in a step-by-step manner, is congruent with the Theory of Transtheoretical Change [27].

### Analysis Method

All primary, secondary, and ancillary analyses are outlined in the published protocol [23]. Sample size calculation and expected effect size are also documented in the protocol [23]. No major changes from the protocol were introduced during study execution. Statistical significance was defined as a *P* value of less than .05 (2-sided test). Effect sizes are reported with 95% confidence intervals. Data were analyzed using SPSS version 20.

#### Primary Analysis

The intention-to-treat principle was followed in the primary analysis. Missing values were managed by the last observation carried forward (LOCF) imputation procedure [28]. The Pearson chi-square test was used to identify any significant difference between the proportion of participants in the control and intervention groups who reported having obtained (or updated) a written AAP during the study.

Binary logistic regression was employed to adjust for potential confounding factors or differences in baseline characteristics that were expected to be predictive of the outcome, including age, gender, past possession of a written AAP, smoking status, medications used for asthma, and past visits to a health care professional for asthma concerns [29].

#### Secondary Analyses

A complete case analysis of secondary outcomes was also conducted using the data of those who completed the poststudy questionnaire and the Pearson chi-square test to identify any significant difference between intervention and control groups.

A comparison was made of the proportion of patients in the intervention and control groups who reported experiencing at least one of the following episodes during the study:

1. Severe asthma exacerbation (as indicated in the Official American Thoracic Society/European Respiratory Society Statement on Asthma Control and Exacerbations) [30];
2. Inadequate asthma control (as measured by Asthma Control Questionnaire [ACQ] score of ≥1.5 in that month) [31];
3. Worsening of asthma that required treatment changes (as measured by a decrease in ACQ score of ≥0.5 between 2 consecutive months) [32]; and
4. Missing one or more days from work or study due to asthma.

#### Ancillary Analyses

Ancillary analyses were conducted to examine reasons for adoption or nonadoption of the intervention. These were conducted using the data of those who completed the poststudy questionnaire or at least one monthly questionnaire. Participant engagement with the intervention was measured via system logs and their perception of the intervention was measured by the Technology Acceptance Model (TAM) instrument [33]. Outcome measures included reasons for obtaining (or not obtaining) a written AAP, participant competing priorities, responses to TAM, and usage of the PCHMS. All measures were reported using descriptive statistics and illustrated with written feedback collected in the poststudy questionnaires. Any recurring patterns or themes reported were emergent from the written feedback from participants. Participant quotes were reported with no alterations.

## Results

### Participant Recruitment, Flow, and Exclusions

**Figure 1:**
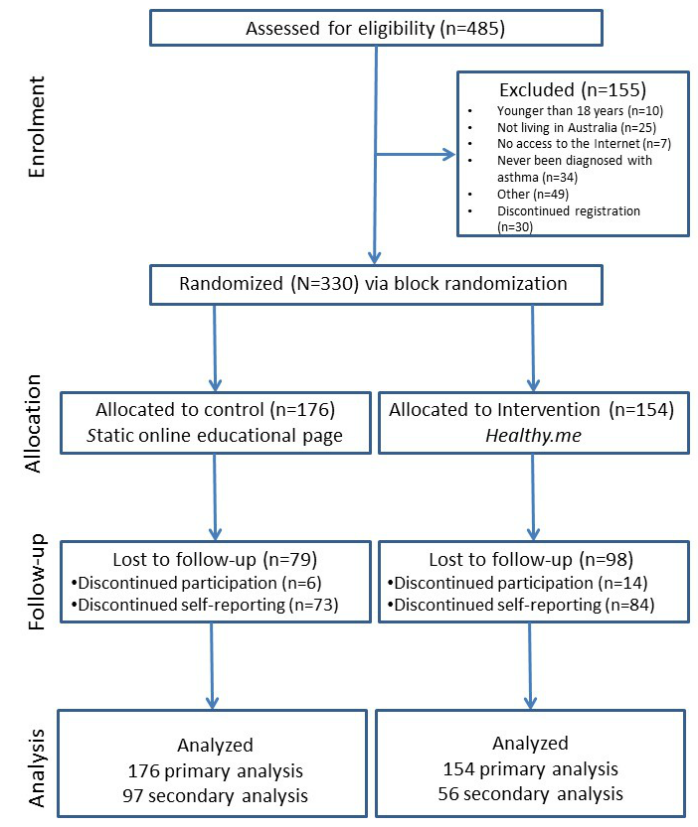
Participant flowchart.

Recruitment was conducted over a period of 5 months between April and August 2013, during which 485 participants were assessed for eligibility (Figure 1). Recruitment was complete in August 2013 and follow-up conducted between April and June 2014. In all, 330 participants were assessed eligible and randomized (intervention: n=154; control: n=176). No participants with available data were excluded from the analyses. No harm or unintended effects were reported by participants during the study.

### Baseline Data

Baseline characteristics were similar for all allocated participants, participants lost to follow-up, and remaining participants (Table 1). The majority of participants were female (control: 79.5%, 140/176; intervention: 80.5%, 124/154), in their late thirties / early forties (control: mean 39, SD 13 years; intervention: mean 40, SD 14 years), and were very familiar with the Internet and social networking sites such as Facebook and Twitter. Asthma-related characteristics, such as smoking status, use of asthma medication, and contact with health care professional for asthma in the past 12 months, were similar to rates identified in a national survey of adults with asthma [22]. However, their rate of possessing a written AAP was higher than the national rate (this study: 38.8%, 128/330 vs Australian Centre for Asthma Monitoring: 21.3% [22]). For those with a written AAP at prestudy, 75.8% (97/128) could not recall when they last obtained or updated it.

**Table 1:** Baseline characteristics of all participants and those lost to follow-up.

Baseline characteristic	All participants		Participants lost to follow-up		Remaining participants	
	Control (n=176)	Intervention (n=154)	Control (n=79)	Intervention (n=98)	Control (n=97)	Intervention (n=56)
Female, n (%)	140 (79.5)	124 (80.5)	59 (75)	80 (82)	81 (84)	44 (79)
Age (years), mean (SD)	39 (13)	40 (14)	37 (12)	36 (12)	41 (14)	46 (14)
Has written AAP (before study), n (%)	71 (40.3)	57 (37.0)	28 (35)	37 (38)	43 (44)	20 (36)
Visited health care professional for asthma in past 12 months, n (%)	142 (80.7)	133 (86.4)	71 (90)	83 (85)	71 (73)	50 (89)
Smoking status, n (%)	16 (9.1)	13 (8.4)	10 (17)	12 (12)	6 (6)	1 (2)
Preventer use in the past 12 months, n (%)^a^	87 (49.4)	78 (50.6)	34 (43)	49 (50)	53 (54)	29 (52)
Reliever use in the past 12 months, n (%)^b^	169 (96.0)	149 (96.8)	76 (96)	93 (95)	93 (96)	56 (100)
Symptom controller use in the past 12 months, n (%)^c^	7 (4.0)	7 (4.5)	4 (5)	5 (5)	3 (3)	2 (4)
Visit social networking sites (eg, Facebook, Twitter) several times a day, n (%)	131 (74.4)	99 (64.3)	67 (85)	66 (67)	64 (66)	33 (59)
Never used the Internet to find health information, n (%)	4 (2.3)	8 (5.2)	2 (3)	6 (6)	2 (2)	2 (4)

^a^ Preventer use: Flixotide, Pulmicort, Qvar, Alvesco, Leukotriene, Singulair, Cromones, Intal, Tilade, Xolair (Omalizumab).

^b^ Reliever use: Ventolin, Asmol, Epaq, Airomir, Bricanyl, Atrovent.

^c^ Symptom controller use: Serevent, Oxis, Fovadile.

### Numbers Analyzed

Analyses of the primary outcome (possession of a written AAP at poststudy) was conducted by intention-to-treat using the data of all 330 allocated participants and the 153 participants who completed the poststudy questionnaire.

We did not apply the intention-to-treat principle to secondary and ancillary outcomes due to the availability of data for analyses. Analyses of secondary outcomes relating to use of the AAP and visits to a health care professional were conducted using the data of 153 participants who completed the poststudy questionnaire. Other study outcomes (ie, asthma exacerbation, asthma control, worsening of asthma, and loss days from work or study) were conducted using the data of 242 participants who completed at least one monthly questionnaire.

### Analysis of Primary Outcome

Analysis of the primary outcome is outlined in Table 2 (more details in Multimedia Appendix 2). There were no significant differences in the proportion of participants who reported having a written AAP poststudy between the intervention and control groups (all participants: χ^2^_1_=0.6, *P*=.43; all participants with imputation method: χ^2^_1_=0.4, *P*=.52; remaining participants at study end: χ^2^_1_=0.9, *P*=.36).

**Table 2:** Analysis of primary outcome by study group (for all participants and remaining participants).

Analysis		n	Has written AAP (poststudy)			Has written AAP (poststudy LOCF^a^)		
			n (%)	χ^2^_1_	*P*	n (%)	χ^2^_1_	*P*
**All participants**				0.6	.43		0.4	.52
	Control	176	38 (22)			66 (38)		
	Intervention	154	27 (18)			64 (42)		
**Remaining participants**								
	Control	97	38 (39)	0.9	.36			
	Intervention	56	27 (48)					

^a^ LOCF: last observation carried forward (imputation method to address missing data).

Binary logistic regression was adjusted for differences in baseline characteristics and potential confounding factors that might influence the primary outcome measure. Only one independent variable made a statistically significant contribution to the regression model: possession of written AAP at prestudy (χ^2^_22_=299.6, *P*<.001). Allocation to the intervention group (OR 0.43, 95% CI 0.15-1.23) did not contribute a significant effect to the proportion of participants possessing a written AAP at poststudy.

### Analysis of Secondary Outcomes

Analyses of secondary outcomes are presented in Table 3 (more details in Multimedia Appendix 2). There were no statistically significant differences in the proportion of participants between the control and intervention groups for any secondary outcome.

**Table 3:** Analyses of secondary outcomes by study group (for remaining participants).

Analysis		Participants, n (%)		χ^2^_1_	*P*
		Control	Intervention		
**Completed poststudy questionnaire (control: n=97; intervention: n=56)**					
	Used AAP more than once during study	20 (21)	11 (20)	0.02	.89
	Visited health care professional for nonemergency asthma	58 (60)	36 (64)	0.1	.71
	Visited health care professional for emergency/urgent asthma	42 (43)	24 (43)	0.003	.96
	Visited emergency department for emergency/unplanned asthma	15 (15)	10 (18)	0.03	.88
	Visited GP or respiratory physician for emergency/unplanned asthma	35 (36)	20 (36)	0.002	.96
**Completed ≥1 monthly questionnaire (control: n=145; intervention: n=97)**					
	Severe asthma exacerbation at least once during study	62 (43)	35 (36)	0.8	.37
	Asthma inadequately controlled at least once during study (as measured by ACQ score ≥1.5)	137 (94)	87 (90)	1.3	.25
	Worsening of asthma that requires a change in treatment (as measured by a decrease of ≥0.5 in ACQ score between 2 consecutive months)	77 (53)	44 (45)	1.1	.29
	Lost days from work or school due to asthma during study	61 (42)	33 (34)	1.3	.26

### Ancillary Analyses

#### Reasons for Not Obtaining a Written Asthma Action Plan

Participant reasons for not obtaining or updating a written AAP during the study are outlined in Table 4. Among control participants, the most frequently cited reason was their “lack of awareness of the plan” (31%, 18/59). Whereas, the reason for PCHMS participants was “none of the above” (33%, 9/27), which included other knowledge, motivation, or belief-related reasons that were not anticipated (such as perceiving the plan to be “irrelevant,” lack of importance placed on asthma, or other life and health priorities which competed for their attention).

**Table 4:** Reasons for not obtaining/updating a written AAP by study group.^a^

Reasons		Participants, n (%)	
		Control (n=59)	Intervention (n=27)
**Knowledge, motivation, or belief-related**			
	I did not know about the existence of AAPs	18 (31)	8 (30)
	I do not believe that a written AAP could be useful to me	10 (17)	4 (15)
	I lacked the motivation to get a written AAP	9 (15)	2 (7)
	I do not know where to get it	7 (12)	1 (4)
	I think the written AAP could be difficult to use	2 (3)	3 (11)
**Other reasons**			
	I did not visit a doctor during the study	13 (22)	6 (22)
	I lacked the time to get a written AAP	10 (17)	2 (7)
	I simply forgot	9 (15)	2 (7)
	It was inconvenient to get it	1 (2)	0
None of the above, please specify^b^		12 (20)	9 (33)

^a^ Participants could select more than one reason.

^b^ Reasons such as perceiving the plan to be “irrelevant,” lack of importance placed on asthma, or other life and health priorities which competed for their attention.

For those who provided further explanation in the poststudy questionnaires, a variety of reasons for not obtaining (or updating) a written AAP were offered.

Participants feeling comfortable with a verbal plan or their own experience in self-management:

My doctor and I have discussed this in detail. No written plan required.

Participant ID 13; accessed PCHMS: once

I am familiar with the steps in a written plan and follow these principles; however, I don’t require an actual hard copy of one, as I am confident in my self-management. Also, the last doctor who tried to force one upon me did not even try to understand my asthma or lifestyle, rather insulting me rude [rudely] and thinking that a generic plan (that included medicine that I do not respond to) was the only way to go. I’m sure he was an exception, but I’m honestly fine with the way I manage my asthma, and when I ask GPs about my medication, it’s rare that there’s anything new going on.

Participant ID 70; accessed PCHMS: once

The poor experience they had with previous written plans / past health care professionals:

I got one a while ago and it was a tick and flick from a drug company and I felt it was useless—gave me nothing more than I know now.

Participant ID 38; accessed PCHMS: once

I have never received one for me though I am a severe asthmatic. My child has received one for whenever he is sick and ends up in hospital. But we have no action plan for either of us for what to do on a normal day and we are feeling unwell with signs and symptoms of asthma.

Participant ID 80; accessed PCHMS: once

Discouragement by health care professionals:

It’s never been offered by doctor.

Participant ID 74; accessed PCHMS: zero times

Doctor told me not to bother.

Participant ID 3; accessed PCHMS: zero times

Competing priorities experienced during the study:

I really didn’t use it very much—not really enough to comment. On a personal note, during the last 12 months, I have been going through a process of appointments and getting my son diagnosed for autism and then ongoing therapies/appointments. I also have 2 other children and am expecting a third plus working part time so I have found adding this extra facet into my life almost impossible. It certainly has nothing against the resource. I have simply been too busy to put the time in and for that I apologize.

Participant ID 51; accessed PCHMS: once

The lack of importance participants placed on asthma:

Complacency—I should I know but having been asthmatic all my life I don’t give it the importance I should.

Participant ID 1; accessed PCHMS: once

I guess I always think it will never get worse...which I know is wrong.

Participant ID 47; accessed PCHMS: twice

A belief that a written AAP is “irrelevant” to their condition:

Look, for someone who has just been diagnosed with asthma or someone quite young, it’s probably great. But for someone like me who has had asthma for over 40 years, has an informal plan of what to do (ie, I know when I need to be on the preventative, what causes it, when I need Ventolin, what to do if I’m having too much Ventolin, etc), it’s not very helpful.

Participant ID 8; accessed PCHMS: twice

I had one, but because my asthma triggers and symptoms and signs change so often they quickly become out of date.

Participant ID 21; accessed PCHMS: twice

Inadequacies of the intervention or asthma content:

Do I have to log in? It would be better if access was open.

Participant ID 41; accessed PCHMS: 4 times

I already understand my asthma. I thought this might contribute to that understanding, but I think it was aimed at a much younger/newer to asthma participant.

Participant ID 8; accessed PCHMS: twice

#### Competing Priorities on Health and Asthma

Participants in both groups were asked to report monthly on their life priorities and the importance they placed on their health and asthma. On a scale from 1 to 10 (where 1 was highest priority and 10 was lowest), participants on average rated health moderately highly (control: mean 3.5, SD 1.9; intervention: mean 3.3, SD 2.1). However, the priority they placed on asthma was not as high (control: mean 4.3, SD 2.2; intervention: mean 3.8, SD 2.3). In fact, asthma was often not a health issue reported by participants that caused them the most concern on a monthly basis.

The average number of life priorities reported by participants was similar in both groups (control: mean 3.0, SD 1.3; intervention: mean 2.7, SD 1.5). These priorities ranged from issues related to work, family/relationship, and money. Health was not always mentioned in this list of priorities. On average, participants reported approximately 2 health issues per month (control: mean 1.9, SD 1.0; intervention: mean 1.5, SD 0.9). These issues are related to a range of bodily systems (eg, cardiovascular, musculoskeletal, psychological, neurological) and not only restricted to the respiratory system.

#### Usage and Perception of Healthy.me

Participant usage and perceptions of the Healthy.me intervention are outlined in Tables 5 and 6. Most participants (80.5%, 124/154) did not access the intervention or accessed it only once (Table 5). Only one person accessed the intervention 10 times or more in this study (Table 5). Because only 30 participants used the website more than once, there was insufficient usage of the online features to make meaningful interpretation of their efficacy. On the TAM scale of 1 (strongly disagree) to 7 (strongly agree), participants indicated, on average, a neutral score (4.7-4.9) for the system’s perceived usefulness and a neutral score for ease of use (4.8-4.9) (Table 6).

**Table 5:** Usage frequency of Healthy.me (n=154 participants).

Usage frequency (times)	Participants, n (%)
0	30 (19.5)
1	94 (61.0)
2-5	27 (17.5)
6-10	2 (1.3)
>10	1 (0.6)

**Table 6:** Perception of the intervention as measured by the Technology Acceptance Model (n=56).^a^

Perception of intervention		Mean (SD)
**Perceived ease of use** ^b^		
	Healthy.me was easy to use	4.9 (1.5)
	I find it was easy to get Healthy.me to do what I wanted it to do	4.8 (1.4)
	It was easy to become confident with using Healthy.me	4.9 (1.4)
**Perceived usefulness** ^b^		
	Managing my asthma through Healthy.me will be beneficial to me	4.7 (1.2)
	The advantages of using Healthy.me to manage my asthma will outweigh the disadvantages	4.9 (1.2)
	Overall, using Healthy.me will help me improve my asthma in general	4.8 (1.2)

^a^ Participants were allocated to PCHMS and completed the poststudy questionnaire.

^b^ Likert scale 1 to 7, where 1=strongly disagree, 4=neutral, 7=strongly agree.

## Discussion

### Principal Findings

Access to the Healthy.me PCHMS did not improve the rate of possession of written AAPs, planned visits to health services for nonemergency asthma management, asthma status, control, or work and study productivity. These results are in stark contrast to earlier trials of the same PCHMS, which showed significant improvements in outcomes associated with consumer behavior change, including influenza vaccination [7] and sexually transmitted infection screening [8]. The negative results also occur in a context where intervention and study designs were in accord with factors typically associated with successful uptake and efficacy of online consumer interventions.

High attrition rates are common in eHealth intervention studies [14], with a recent systematic review revealing that completion of protocol rates for depression sites ranged from 43% to 99% [34]. This study suffered from moderate to high rates of attrition in the intervention (64%) and control (45%) groups. A previous study of Healthy.me did not experience this degree of attrition [7]. However, that study was conducted in 2009, its duration was shorter, and the mean participant age was 26.2 years compared to 40 years among those allocated to the intervention in this RCT [7].

### Utilization and Benefit

In decision theoretic terms, the expected utility of any eHealth intervention is a product of the utility or benefit of each individual interaction with the system to the user and the number of times the interaction takes place [35]. Systems that engage their users and, as a result, are used frequently are theoretically more likely to deliver benefit. This is reflected in the research evidence, where consumer eHealth systems seem to demonstrate a clear dose-response relationship between use and benefit [35].

In this trial, some participants suggested that they saw little benefit in using the system, either because they or their health professionals saw little value in having an AAP, because asthma management was not a major priority in their life compared to other competing priorities, or that they have already developed their own strategies to manage the condition and needed no further assistance. Perhaps as suggested by some participants, the intervention would be more helpful for those who are newly diagnosed with asthma.

### Comparison With Prior Work

A systematic review of PHRs used for chronic conditions found that unless a system clearly assisted consumers in self-management tasks, they were unlikely to be successful [36]. This benefit might come from tracking important parameters to control an illness, such as blood pressure or glucose levels, or by delivering feedback when changes to management are needed. That review identified diabetes, hypertension, asthma, HIV, fertility management, glaucoma, and hyperlipidemia as having the most evidence for PHR benefit. However, only one study in that review actually included asthma patients and these were grouped together with other patients who had diabetes or hypertension [36]. The only outcome measure was patient activation, and asthma patients represented only 7% of the sample, providing weak evidence of PHR benefit in asthma management outcomes.

Recent systematic reviews concluded that although there is evidence that some digital interventions are associated with positive asthma self-management outcomes [37,38], most interventions do not use behavioral change theory, clinical guidelines, and/or assessment tools to inform their design [37]. A Cochrane review on smartphone apps for asthma concluded there is currently lack of evidence to advise clinical practitioners, policy makers, and the general public on ways to implement these interventions for asthma self-management programs [39]. Relevant to the AAP, a theoretical model has proposed 4 elements that are essential in facilitating the “right” contexts between patients and professionals, but few studies have used all these elements in their implementation [40].

Our own earlier trials of this intervention focused on supporting preventive health tasks. A trial aimed at encouraging influenza vaccination demonstrated a significant doubling in vaccination rates, most likely because the system allowed easy and immediate access to booking a vaccination with a primary care center, for a condition where seasonality and acting in a timely manner is important [7]. Similar benefits were demonstrated when the system was targeted at increasing screening rates among young adults for sexually transmitted infections, where use of an online booking system may have additionally reduced any sense of stigma associated with making a decision to act [8].

### Lessons Learned

Although the lack of uptake of eHealth interventions is a widely known phenomenon [13-15], the literature on negative findings in this field is still scarce. Our study provides a number of lessons:

1. *Consumers must perceive the need for assistance with a task.* Even though the research evidence clearly demonstrates the value of an AAP, its low level of adoption in the population and the commentary received in this study suggests that at least some adults with asthma either do not agree or have yet to be convinced.
2. *Consumers must assign priority to the tasks supported by the intervention.* Participants in this study assigned a low priority to their asthma management compared with other life priorities. The mean age of participants in this study was 40 and most reported living with multiple competing priorities (eg, work commitment, lack of time) and other health concerns (eg, multimorbidity).
3. *The cost of adoption of the intervention must be lower than the benefit.* Our PCHMS was a stand-alone system that did not integrate into other apps participants might already have been using, such as diaries and social media. It consequently required additional effort to use. A substantial number of participants were recruited via a Facebook social network related to asthma and used Facebook several times a day. These individuals may have had higher expectations of the intervention regarding the degree of system integration, content, social network size, and the overall “polish” of the system.
4. *Outcome measures must be relevant to consumers and providers.* Although the primary outcome used in this RCT—possession of an up-to-date written AAP—is an indicator of recommended care for asthma [41], it is essential to consider how relevant and important the outcome measure is to *both* consumers and providers. Although there is evidence supporting the efficacy of the written AAP, there is possibly the misconception that it is only useful for those newly diagnosed with asthma. Perhaps there needs to be more emphasis on uncovering how relevant an outcome measure is to both consumers and providers before attempts are made to influence behavior change.

### Implications for Consumer eHealth Design

#### Design for Attrition

Although current evidence advocates the importance of having a theoretical basis to direct behavioral changes, it is equally important to consider whether such theories can be used to minimize participant attrition. For example, identifying early on those who are truly uninterested and focus instead on those who are likely to continue could potentially reduce participant attrition [14]. Perhaps all interventions should be designed with a plan to minimize participant dropout before commencing participant recruitment.

#### Design for Implementation

Studies have confirmed once again that implementation uptake is often the biggest challenge in any eHealth project, both for consumers and clinicians. Trials that focus on implementation of asthma interventions are emerging in clinical settings [42,43]. Yet, implementation strategies that consider consumer settings, their comorbidities, and their competing demands are lacking. Understanding how these consumer factors affect the uptake of an intervention is important. A recent review on digital interventions for asthma concluded patient perspectives are often largely ignored [38]. Perhaps the next generation of digital intervention should incorporate consumer-clinician implementation strategies at the core of every digital intervention design.

#### Design for Context

Rather than attempting to “perfect” the design of an intervention to exist on its own, interventions should be designed for the context. When designing an intervention for consumers and patients, it is important to identify early on whether the intervention should focus on task support or on belief change. Moreover, research should focus on how we can design consumer eHealth interventions that are integrated in health care settings and/or how such interventions would function in the consumer circle of care (eg, caregivers).

### Limitations

Study strengths include nationwide recruitment, use of recommended care indicators for outcome measures, and triangulation of participant feedback with quantitative results.

Notable limitations of this study include the gender and age distribution of participants, the attrition rate, and the use of self-reported data. The majority of participants were female in their late thirties / early forties and it is possible this population sample behaved differently than a more representative sample.

Participants had a higher rate of AAP possession than reported in other studies. As a result, as a cohort, they may already be better engaged and confident in their self-management and less likely to benefit from the intervention compared to the population average, reducing the potential effect size. Further, because the outcome measure was focused on having an up-to-date written AAP that was updated by a clinician (eg, once a year), we may have missed some participants as the study duration was only 12 months. Future studies should consider extending the trial period to more than 12 months.

Our primary recruitment strategy is online, which has a number of limitations, such as high rates of attrition. More effective recruitment could potentially result when it is channeled through influencers such as health care providers or with the encouragement of caregivers who help patients to deal with issues every day. However, this is an intervention designed primarily for consumers, to be delivered online, thus it is important that there is a direct channel to recruit consumers who are already online.

### Conclusions

Consumers are increasingly turning to the Internet and social media for health advice, yet we still do not fully understand why some online interventions work and others do not. In this study, participant goals were poorly aligned with the clinical goals of the system despite there being clear evidence underpinning these latter clinical goals. It may be that a different approach is required in the domain of asthma management in adults, at least as far as AAPs are concerned, focusing not so much on task support as on belief change. More generally, researchers should not feel discouraged to publish negative findings because in failure many significant lessons can be learned.

## Multimedia Appendix 1

Use of Health Belief Model (HBM) in Asthma Patient Journeys.

## Multimedia Appendix 2

Utilization of Personally Controlled Health Management System (PCHMS).

## Abbreviations

AAP: asthma action plan
ACQ: Asthma Control Questionnaire
HBM: Health Belief Model
LOCF: last observation carried forward
PCHMS: personally controlled health management system
PHR: personal health record
RCT: randomized controlled trial
TAM: Technology Assessment Model
