# Supplementary material for: “Why Didn’t it Work?” Lessons From a Randomized Controlled Trial of a Web-based Personally Controlled Health Management System for Adults with Asthma
Source: J Med Internet Res. 2015 Dec 15;17(12):e283. doi: 10.2196/jmir.4734 (PMC4704895; doi:10.2196/jmir.4734)
Supplement: Supplementary file 1 [file jmir_v17i12e283_app1.pdf]

Appendix 1: Use of Health Belief Model (HBM) in Asthma Patient Journeys

The three asthma journeys are based on the Health Belief Model, and incorporate its constructs of perceived susceptibility, severity, barriers, benefits, and self-efficacy. These constructs are incorporated into each stage of the asthma journey.

The first journey aims to increase the participants' perceived susceptibility to, and severity of, asthma, through increasing their asthma-related knowledge and understanding of prevalence, risk factors, and indications/recommendations for obtaining a written AAP. Specifically, this journey addresses what is a written AAP, and why it is important to have one (regardless of whether one's asthma is well controlled).

The second journey attempts to directly address many of the perceived barriers to obtaining a written AAP screening, including those regarding costs, time, and finding an appropriate healthcare professional.   Specifically, this journey outlines the steps in getting a plan (e.g. preparing questions to ask the healthcare professional, and highlighting issues that one needs to inform the healthcare professional about asthma).

The third journey highlights the importance of the written AAP by offering tips to people living with asthma in different circumstances (such as the importance of getting an influenza vaccine for winter); thereby enhancing self-efficacy. Each tip was delivered to participants by email on a monthly basis, and this formed part the participant engagement strategy to minimize attrition rate during the study.

Overall, the perceived benefits of obtaining a written AAP, and its presentation as a positive social norm, are appropriately incorporated and reinforced in all three journeys. Additionally, the informational 'journey' content was designed to be succinct, focusing on the main identified barriers and facilitating factors for getting a written AAP in adults with asthma.
