# Supplementary material for: “Why Didn’t it Work?” Lessons From a Randomized Controlled Trial of a Web-based Personally Controlled Health Management System for Adults with Asthma
Source: J Med Internet Res. 2015 Dec 15;17(12):e283. doi: 10.2196/jmir.4734 (PMC4704895; doi:10.2196/jmir.4734)
Supplement: Supplementary file 2 [file jmir_v17i12e283_app2.pdf]

Appendix 2: Utilization of Personally Controlled Health Management System (PCHMS)

Table 1: Baseline characteristics by PCHMS Utilization

**Baseline characteristic**	**PCHMS Utilization (n=154 participants)** [1]	
	Zero (n=58)	Once or more (n=96)
Female % (n)	46 (79.3%)	78 (81.3%)
Mean age (SD), years	39.9 (13.52)	39.3 (13.77)
Has written AAP (before study), % (n)	25 (43.1%)	32 (33.3%)
Visited healthcare professional for asthma in past 12 months, %	51 (87.9%)	82 (85.4%)
Smoking status, %	52 (89.7%)	85 (88.5%)
Preventer use in the past 12 months, % [a]	37 (63.8%)	41 (42.7%)
Reliever use in the past 12 months, % [b]	54 (93.1%)	95 (99.0%)
Symptom controller use in the past 12 months, % [c]	49 (84.5%)	86 (89.6%)
Visit social networking sites (e.g. Facebook, Twitter) several times a day	41 (70.7%)	58 (60.4%)
Never used the Internet to find health information	5 (8.6%)	3 (3.1%)

[1] Out of 154 participants who were allocated to PCHMS

Table 2: Primary and secondary outcomes by PCHMS Utilization

	**PCHMS Utilization (n=154 participants)** [1]	
**Primary outcome**	Zero (n=58)	Once or more (n=96)
Has written AAP (post-study LOCF [a])	26 (44.8%)	38 (39.6%)
	**PCHMS Utilization (n=56 participants)** [2]	
**Secondary outcome**	Zero (n=10)	Once or more (n=46)
Used AAP more than once during study	2 (20%)	9 (19.6%)
Visited healthcare professional for non-emergency asthma	6 (60%)	30 (65.2%)
Visited healthcare professional for emergency/ urgent asthma	6 (60%)	24 (52.2%)
• Visited Emergency Department for emergency/ unplanned asthma	2 (20%)	8 (17.4%)
• Visited GP or respiratory physician for emergency/ unplanned asthma	4 (40%)	16 (34.8%)
**Perception of PCHMS**		
Perceived ease of use, mean (SD) [3]		
• Healthy.me was easy to use	5.3 (2.00)	4.8 (1.42)
• I find it was easy to get Healthy.me to do what I wanted it to do	5.3 (2.00)	4.7 (1.28)
• It was easy to become confident with using Healthy.me	5.4 (2.07)	4.8 (1.21)
Perceived usefulness, mean (SD) [3]		
• Managing my asthma through Healthy.me will be beneficial to me	5.7 (1.34)	4.5 (1.07)
• The advantages of using Healthy.me to manage my asthma will outweigh the disadvantages	5.7 (1.34)	4.7 (1.07)
• Overall, using Healthy.me will help me improve my asthma in general	5.6 (1.71)	4.6 (0.95)

[1] Out of 154 participants who were allocated to PCHMS
[2] Out of 56 participants who were allocated to PCHMS and completed the post-study questionnaire
[3] Likert scale 1 to 7, where 1=Strongly disagree, 4=Neutral, 7=Strong agree
